# Interaction of viral oncogenic proteins with the Wnt signaling pathway

**DOI:** 10.22038/IJBMS.2018.28903.6982

**Published:** 2018-07

**Authors:** Sayyad Khanizadeh, Banafsheh Hasanvand, Hamed Esmaeil Lashgarian, Mohammad Almasian, Gholamreza Goudarzi

**Affiliations:** 1 Hepatitis Research Center, Lorestan University of Medical Sciences, Khorramabad, Iran; 2 Department of Virology, School of Medicine, Lorestan University of Medical Sciences, Khorramabad, Iran; 3 Department of English Language, School of Medicine, Lorestan University of Medical Sciences, Khorramabad, Iran; 4 Department of Microbiology, School of Medicine, Lorestan University of Medical Sciences, Khorramabad, Iran

**Keywords:** β-catenin, Canonical pathway, Carcinogenesis, Oncogenic viruses, Wnt signaling

## Abstract

It is estimated that up to 20% of all types of human cancers worldwide are attributed to viruses. The genome of oncogenic viruses carries genes that have protein products that act as oncoproteins in cell proliferation and transformation. The modulation of cell cycle control mechanisms, cellular regulatory and signaling pathways by oncogenic viruses, plays an important role in viral carcinogenesis. Different signaling pathways play a part in the carcinogenesis that occurs in a cell. Among these pathways, the Wnt signaling pathway plays a predominant role in carcinogenesis and is known as a central cellular pathway in the development of tumors. There are three Wnt signaling pathways that are well identified, including the canonical or Wnt/β-catenin dependent pathway, the noncanonical or β-catenin-independent planar cell polarity (PCP) pathway, and the noncanonical Wnt/Ca2+ pathway. Most of the oncogenic viruses modulate the canonical Wnt signaling pathway. This review discusses the interaction between proteins of several human oncogenic viruses with the Wnt signaling pathway.

## Introduction

Viruses are known as one of the major agents of human cancers (1). Risk factors, such as chemical agents, radiations, mutations, genetic factors, and infection with oncogenic viruses can be considered as significant predisposing factors in carcinogenesis (2). Both DNA and RNA viruses in different virus families can cause cancer, however, oncogenic viruses are mostly DNA viruses (1). Oncogenic viruses have characteristics that can help them in the process of tumorigenesis, including the fact that these viruses usually cause chronic infections and carry oncogenes (2). The protein products of oncogenes have a direct impact on the process of tumorigenesis. In addition, nucleic acids and viral sequences are always present in cancerous cells and can be detected (3). In the process of carcinogenesis in a cell, there are various mechanisms involved, but it can be said that, in general, viral oncoproteins can directly or indirectly play a part in the activation of the signaling pathways in the carcinogenesis process through affecting and involving various cellular pathways, especially the regulatory and controlling pathways of the cell cycle. Additionally, viral oncoproteins can disrupt the cell cycle and the cellular regularity, which results in cellular transformation by producing cellular analogs such as anti-apoptosis proteins, inducing cellular proto-oncogenes, targeting cell cycle checkpoints, (eg, P53, RB, PP2A) and various transcription factors (4).

***Review***

*Oncogenic viruses and cellular pathways*

Viral Carcinogenesis is a time-consuming process in which various cellular pathways are involved (3). Many of oncogenic viruses establish a persistent infection, during the chronic stage of diseases, which enables them to subvert or activate these pathways (3, 4). The tumorigenic strategy of oncogenic viruses is similar so that these viruses target key cellular signaling pathways. Most oncogenic viruses modulate the cell cycle progression to rescue cells from apoptosis. The cellular targets for viral oncogenic proteins are generally the vital transcriptional factors involving tumorigenesis, such as pRb, c-myc, NF-κB, p53, AP-1 (4, 5). In certain cancers, such as cervical cancer and HPV, viral infection causes a phenomenon called “hit and run”, in which the infection acts as a triggering effective blow in the tumorigenic process, and subsequently, cellular carcinogenesis pathways are activated. Carcinogenesis is driven by genetic and epigenetic changes that allow the cells to have uncontrolled growth and escape mechanisms that naturally regulate and differentiate cells (5). Many of these changes occur in cell signaling pathways that control growth, division, death, differentiation, and cell migration. This scenario results in alterations in the microscopic situation of the tumor, angiogenesis, inflammation, and mutations that convert proto-oncogenes to oncogenes (6).

*Wnt signaling pathways*

The Wnt signaling pathways are a group of signal transmission pathways into the cell, which are made of proteins that pass signals into the cell via cell surface receptors. The Wnt proteins comprise a diverse range of proteins (7). There are about 16 types of these proteins in humans and mice, and they are also found in other organisms such as Xenopus, Zebrafish, Drosophila, and many other animal species (8). There are three Wnt signaling pathways that are well characterized. These pathways include the canonical Wnt pathway, the noncanonical planar cell polarity (PCP) pathway, and the noncanonical Wnt/Ca^2+^ pathway (7). As their names suggest, these pathways belong to either the canonical or the noncanonical categories. The difference between these two groups is that the canonical pathway involves the β-catenin protein, while the noncanonical pathway acts independently of β-catenin (9).

*Activation of Wnt signaling pathways*

The activation of Wnt signaling pathways is initiated via binding a Wnt protein ligand to a Frizzled (Fz) family receptor that transduces the corresponding signal to the phosphoprotein Dishevelled (Dsh) protein inside the cell cytosol (8, 9). Dsh is a common mediator between the three Wnt signaling cascades. Typically, in the absence of Wnt proteins, β-catenin is not able to accumulate within the cytoplasm; because destruction complex (CK1α, APC, Axin, and GSK3) would target it, and subsequently would be digested via the proteasome. In the canonical pathway, activation is initiated via interaction between Wnt and Frizzled (Fz) receptor, induced by RSPO molecule and recruitment of Dsh and destruction of CK1, APC, AXIN, and GSK3β complex. This process leads to the accumulation of β-catenin in the cytoplasm. Subsequently, β-catenin translocates to the nucleus (10). In the nucleus, β-catenin binds TCF/LEF promoters that are driving expression of target genes, including AXIN2, C-myc, and CCND1 (9). The activation of β-catenin independent pathways including planar cell polarity (PCP) pathway and the calcium pathway is initiated via the ROR and different Wnt/FZD bindings and Ryk receptors. The calcium pathway contributes to the activation of Ca2+/CAMKII, cell adhesion, NFAT, and CREB. The activation of the PCP pathway involves molecules such as Rac, RhoA, GTPases, and the JNK cascade. This results in processes including apoptosis, proliferation, and differentiation. In addition to their independent functions, these pathways overlap biological cellular processes and can also affect other cellular pathways. The Wnt signaling pathways are involved in important cellular processes such as embryonic development, axis patterning, cell fate specification, cell proliferation, insulin sensitivity, and cell migration, all of them having clinical implications and are considered as one of the most important and central pathways for carcinogenesis signaling (10). From the beginning of the discovery and identification of these pathways, when it was observed in a mouse model study that the first protein detected in this pathway (Wnt1) acts as a proto-oncogene in breast cancer, it was recognized that these pathways are related to cancer, especially breast cancer (11). In addition to breast cancer, the Wnt signaling pathways are associated with the formation and progression of other malignancies and cancers such as colorectal, melanoma, liver, prostate, lung, and lymphoma malignancies (12). 

*Epstein bar virus (EBV) and Human herpesvirus-8(HHV-8)*

**Figure 1:**
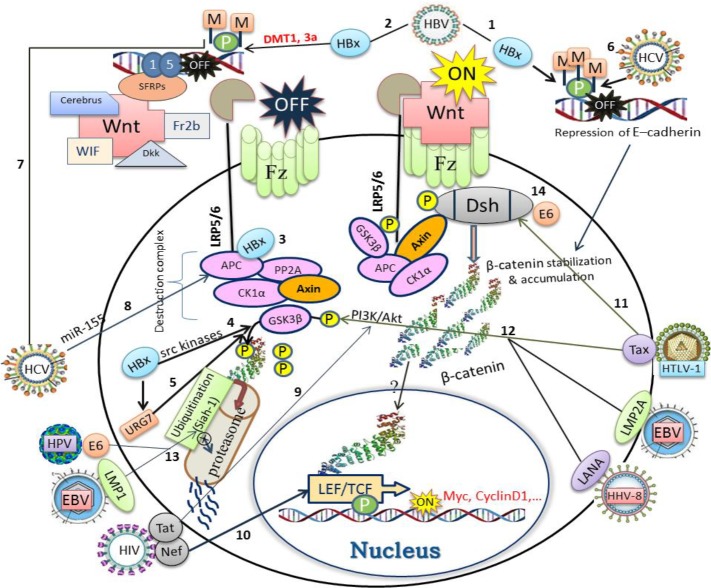
The schematic representation for the possible interaction of viral oncogene proteins with various levels of Wnt/β-catenin cell signaling cascade. (HBV): (1) hypermethylation of E-cadherin, (2) SFRP1 and SFRP5 promoters via HBx protein; (3) dislocating of β-catenin from the destruction complex by binding to APC; (4) HBx-mediated suppression of GSK3β by src kinases, (5) overexpression of URG7 and the final inactivation of GSK3β. (HCV): (6) HCV core protein mediated-hypermethylation of E-cadherin promoter down-regulates E-cadherin expression and thereby β-catenin accumulation; (7) silencing of SFRP1expression by hypermethylation of its promoter; (8) upregulation of miR-155 gene and targeting of APC. (HIV): (9) Tat protein activates PI3K/AKT signaling pathway and inactivates GSK3β; (10) Nef protein compete for the similar site occupied by LEF/TCF on β-catenin. (HTLV-1): (11) Tax protein interacts with DAPLE (disheveled-associating protein) to trigger the canonical Wnt pathway; (12) Tax raise PI3K/Akt activity, resulting in the phosphorylation and inactivation of GSK3β. (EBV): (12) LMP2A activates PI3K/Akt pathway, resulting in the phosphorylation and inactivation of GSK3β. (13) LMP1 inhibits Siah1 (an E3 ubiquitin ligase), which is involved in ubiquitination and proteasomal degradation of β-catenin; (HPV): (13) E6 oncoprotein inhibits Siah1, which is involved in ubiquitination and proteasomal degradation of β-catenin; (14) E6 binds to Dsh and disrupts the destruction complex (β-catenin stabilization). (HHV-8): (12) LANA protein promotes PI3K/Akt activity, resulting in the phosphorylation and inactivation of GSK3β

EBV and HHV-8 belong to the *Herpesviridae* family. These viruses have a coding-genomic double-stranded DNA for enzymes involved in replication, repair, and biosynthesis of viral nucleic acid. EBV and HHV-8 can establish latent infections within the lymphoid cells and tissues that can be activated when the host immune system is suppressed (13). EBV or human herpesvirus 4 (HHV-4), is a ubiquitous virus, so that, more than 90% to 95% of adults around the world are infected with it. This virus infects the B lymphocytes and the epithelial cells. During primary infection, it causes acute infectious mononucleosis and during persistent infection, it is associated with Burkitt lymphoma, nasopharyngeal carcinoma (NCP), Hodgkin’s disease, non-Hodgkin lymphoma, and gastric carcinoma, especially in immune-deficient individuals (14). Many studies have documented that among EBV proteins (Table 1), two latent membrane proteins (LPM1 and LMP2) play an important role in the EBV pathogenesis. LMP2A is critical for the efficient activation, survival, and proliferation of EBV-infected B cells; it can affect the efficient long-term growth of B cells (15). This protein can interact with various cellular pathways including activation of the canonical Wnt pathway (Figure 1). Researchers have demonstrated that LMP2A activates β-catenin signaling in epithelial cells, their results have shown that the ITAM motif of EBV-LMP2A via interaction with Act and PI3K signaling leads to nuclear accumulation of β-catenin and targeting of the Wnt signaling pathway (20). Additionally, in an *in vitro* study on the several EBV-positive tumor cell lines including lymphoblastoid cell lines (LCL), compared to EBV-negative lines, it was shown that in contrast to epithelial cells, β-catenin accumulates in the cytoplasm but not in the nucleus (21). Further, the studies also suggested that LMP2A via upregulation of Wnt5 can activate non-canonical Wnt signaling. Reports have shown that LMP1 can lead to inhibition of SIAH1 (is involved in ubiquitination and proteasome-mediated degradation) expression in B lymphoma cells and the upregulation of β-catenin. Furthermore, this protein increases cytoplasmic β-catenin levels and induces hyperplasia in epithelial cells (18). In addition, reports have shown that EBV-miR-BARTs via their negative effects on the Wnt signaling protein inhibitors are involved in the metastasis and progression of nasopharyngeal carcinoma (64). HHV-8 or human herpesvirus type 8, known as the Kaposi sarcoma tumor (KSHV) agent in HIV positive individuals, is a human herpesvirus associated with diseases such as the malignancy of the B-cell lymphoproliferative disorder, primary effusion lymphoma and the multi-centric Castleman’s disease (65). Studies have shown that HHV-8 can interact with the Wnt signaling pathway (16, 18, 64, 65), and this interaction is effective in the HHV-8 carcinogenesis and latent infection (16). Observations have shown that one of the HHV-8 proteins known as LANA, acts as an oncoprotein and inhibits p53, and retinoblastoma protein also causes nuclear β-catenin accumulation through interaction with GSK3β (Figure 1) (17). In these reports LANA trapping of GSK3β in the nucleus was observed resulting in cytoplasmic depletion of GSK3β, subsequently GSK3β entering the nucleus, leading to accumulation of β-catenin and activation of downstream the Wnt signaling transcriptional responses. Additionally, other studies have shown that HHV-8 may increase the expression of β-catenin and Wnt7a in epithelial cells via coding the chemokine receptor (vGPCR) homolog and interaction with the PI3K/Akt pathway (16-18, 64, 65).

*Hepatitis B virus (HBV) and Hepatitis C virus (HCV)*

HBV and HCV are well-known viral infections in all over the world. The worldwide prevalence of chronic HBV infection is between 240 and 350 million and more than 170 million are chronically infected with HCV. The chronic infections caused by these viruses after 2 to 3 decades can lead to dangerous clinical consequences such as fibrosis, cirrhosis, and hepatocellular carcinoma (HCC) (66-68). HBV is a member of the *Hepadnaviridae* family, containing a partially double-stranded DNA genome and several structural and soluble proteins. One of the most important proteins of HBV is HBx, considered as an oncoprotein because of its carcinogenic characteristics (69, 70). Several studies have demonstrated an association between the HBx oncoprotein and the HBV surface protein (HBsAg) with the Wnt signaling pathway, especially activation of canonical Wnt/β-catenin signaling (Figure 1). It has been demonstrated that the HBx protein can stimulate the expression of the MYC and CCND1 (coding beta-1-catenin gene) genes, the targeted genes in the Wnt pathway, and nuclear ß-catenin aggregation in animal models (22, 71, 72). A study has shown that the genetic/epigenetic aberration by HBV infection in the CTNNB1 gene and the mutations of APC and AXIN1 genes play a crucial role in HBV-associated hepatocellular carcinoma (23). Researchers have reported that the HBx oncoprotein was associated with the hypermethylation of SFRP1 and SFRP5 proteins in HCC/HBV positive patients *in vitro*, which results in a substantial increase in the expression of the Wnt signaling pathway (24). In other studies, the association between the HBV proteins and the proteins of Wnt signaling pathway has been investigated (25, 73, 74). The results of various studies have shown, HBx is associated with repression of SFRP1 and SFRP5 (two Wnt signaling antagonists), disintegration of the E-cadherin complex with β-catenin, binding to APC and displacing β-catenin from the destruction complex, suppression of GSK3β activity via activation of src kinases, and upregulation of expression of URG7(a protein with anti-apoptotic effects), which in turn activates the Wnt signaling either by transactivation of β-catenin or inactivation of GSK3β. The presence of HBV proteins leads to up-regulation of LEF-1, CCND1, and MYC; also HBcAg protein can be bound to 41 Wnt pathway gene promoters in an unknown mechanism, *in vitro* (18, 25, 26). Most studies and reports have confirmed the association between the HBx protein and Wnt signaling pathway (25, 26, 74). Some studies have been conducted on HBV and the Wnt signaling pathway using the immunohistochemical method in HBV-positive HCC patients, indicating β-catenin accumulation as an indicator of the activation of the Wnt signaling pathway (75, 76). Researchers have inserted the HBX gene into LINE1 elements in host cell chromosomes in HBV-positive HCC patients, resulting in the activation of the Wnt signaling pathway (77).

 HCV is the most common cause of chronic liver infection, classified in the *Flaviviridae* family. HCV contains several structural proteins (core, E1, and E2) and non-structural proteins (NS1, NS2, NS3, NS4, and NS5), associated with the pathogenesis of HCV (27, 78). Early studies showed Wnt signaling pathway is a key pathway in HCV-positive HCC (Figure 1), the function of SFRP4 and RUNX3 as Wnt inhibitors in HCV infection being specific (28-30, 34). Epigenetic changes such as the methylation of the SFRP2 gene have been observed in the pathogenesis of HCV positive HCC patients (31). Quan *et al*. (2014) have suggested that the HCV core protein increased the expression of Wnt signaling proteins, via hypermethylation of the SFRP1 promoter, resulting in epigenetic silencing of SFRP1 expression (32). HCV proteins can affect the expression of E-cadherin via modulation of the Wnt signaling and reducing E-cadherin (a cell adhesion molecule) expression at the transcriptional level. The transfection of HCV core protein in human hepatoma cell lines upregulated Wnt1 and the targeted genes of this pathway (79). HCV core protein is effective in activating β-catenin/Tcf-4-dependent expression and also enhances β-catenin expression level and nuclear stabilization of the protein, additionally core protein induces gene expression of canonical Wnt ligands (Wnt-proteins and LRP5/6 co-receptors) (33). 

In a preliminary study, it was shown that the NS5A protein as an important viral non-structural protein could result in β-catenin stability in the nucleus and the activation of the canonical Wnt pathway (29, 30, 38). This protein can also activate the expression of c-myc proto-oncogene (38). Furthermore, HCV proteins can interact with the cellular microRNA network and cause the modulation of microRNA expression and cellular pathways (35, 36, 39). It was found that HCV infection stimulates inflammation and the Wnt signaling pathway through induced expression of miR-155 gene, followed by the upregulation of CCND1, MYC, BIRC5, and nuclear β-catenin. It was observed that APC (a tumor suppressor gene that inactivates Wnt/β-catenin pathway) is the direct biological target of miR-155 (37). 

**Table 1:** Effect of viral oncogenic proteins on the Wnt signaling pathway

Virus	Viral oncogenic protein	Molecular mechanism	References
HHV-8	LANA	- Inhibition of p53 and retinoblastoma protein. Accumulation of nuclear β-catenin via interaction with GSK3β. vGPCR-mediated up-regulation of β-catenin and Wnt7a in epithelial cells	(16, 17)
EBV	LMP1	- Inhibition of SIAH1 expression in B lymphoma cells and up-regulation of β-catenin. Increased cytoplasmic β-catenin and hyperplasia induction of epithelial cells.	(18)
	LMP2	-Inhibition of epithelial cell differentiation. Accumulation of β-catenin is in the cytoplasm. The methylation of Wnt signaling proteins. Up-regulated viral miRNAs that target Wnt signaling. Expressed EBV- miR‐BARTs that target Wnt signaling.	(19-21)
HBV	HBx	-Disintegration of the E-cadherin complex with β-catenin, binding to APC and displacing β-catenin from the destruction complex, suppression of GSK3β activity, modulation of CTNNB1, APC, and AXIN1 gene expression. Silencing of SFRP1 and SFRP5 proteins. Insertion of the HBX gene into LINE1 elements and activation of Wnt signaling pathway.	(22-24)
	HBc and other proteins	- Upregulation of the LEF-1, CCND1, and MYC genes, HBc can be bound to 41 Wnt pathway gene promoters.	(18, 25, 26)
HCV	Core	-Induced expression of Wnt signaling proteins via SFRP1 hyper-methylation. Downregulation of the Wnt gatekeepers such as DKK1, SFRP3 and SFRP5.	(27-34)
	NS5A	-Activation of the Wnt pathway via beta-catenin nucleus stability. Expressed c-myc proto-oncogene and DNA damaging. Modulation of cellular microRNA expressions such as miR-155. Epigenetic changes including Methylated DKK1 and SFRP2 genes, SFRP4 and RUNX3 genes in HCV positive subjects	(35-39)
HTLV	Tax	- Nuclear stability of beta-catenin. Activation of the Wnt pathway through interaction with the Wnt proteins such as Wnt5a and leucine-rich disheveled (Dvl)-associated protein. Inhibition of GSK3β	(40-42)
	HBZ	- Upregulation of the DKK1 gene in epithelial cells. Upregulating the Wnt5a gene	(41)
HIV	Tat	-Interaction with TCF4 and inhibition of Wnt signaling. Induced DKK gene expression.	(43, 44)
	Nef	-Interaction with β-catenin proteins and T-cell transmigration.	(45, 46)
	gp120	-Upregulating BDNF expression in BV2 cells through the Wnt/β-catenin signaling.	(46)
HPV	E6 and E7	- Interaction with Rb and P53 proteins. Stimulating or enhancing Wnt/β-catenin signaling. Inducing β-catenin-TCF/LEF-mediated transcription. Upregulating proto-oncogenes MYC and CCND1. Dysregulation of the Wnt pathway via modulation of MYC, FZD, DKK, and WNT16 genes.	(47-53)
CMV	US28	- Promoted intestinal adenomatosis and CCND1 and nuclear β-catenin expression. Increased β-catenin independent of the Wnt pathway. Coding viral miRNAs that target the β -catenin. Induced Wnt2, and WISP2 and reduced Wnt5a, LRP6, CCND1, MYC, and DKK gene expressions.	(54-58)
Adenovirus	E1	-Dysregulation of the Wnt signaling pathway in fibroblast cells.	(59-61)
Enterovirus	Type 1	-Modulation of the Wnt pathway through regulated expression of miRNAs.	(62)
Coxsackievirus		Targeting LRP6 and WRCH1 and promotion of β-catenin degradation.	(63)

*Human T-cell lymphotropic virus (HTLV) and Human Immunodeficiency Virus (HIV)*

Retroviruses are a large viral carcinogenic family that can cause tumors in both humans and animals, therefore there are carcinogenic viral prototypes in this family (81-84). Human T-cell lymphotropic virus (HTLV) is one retrovirus that tends to affect human mature T lymphocytes. Several types of HTLV have been identified. In humans, HTLV1 is predominantly responsible for adult CD4+ T cell leukemia (85, 86). HTLV1 has an important viral protein called Tax, associated with many carcinogenic characteristics of the virus (40, 87-89). Reports indicate that Tax can interact with the Wnt signaling pathway (Figure 1) and plays a role in the nuclear stability of beta-catenin (41, 42, 90). Researchers have found that the HTLV-1 Tax via activating the CREB signaling pathway can activate the PI3K/Akt pathway, subsequently phosphorylated Akt inhibits GSK3β. Suppression of GSK3β prevents proteasomal degradation of β-catenin, resulting in translocation of β-catenin to the nucleus and binding to the Tcf promoter (90). Also it is observed, that Tax could activate the canonical Wnt signaling via interaction with a Wnt pathway-related protein, the leucine-rich disheveled (Dvl)-associated protein (a high-frequency leucine residue) (DAPLE), but result in downregulation of two transcription factors (TCF1 and LEF1), mainly expressed in T cells, suppress the trans-activating ability of Tax (88, 89). In other studies, it has been found that the HTLV-1 βZIP factor (HBZ) actually activates a noncanonical Wnt pathway via interaction with Wnt5a, which is an antagonized the canonical Wnt signaling pathway, such that knocking down Wnt5a in HTLV-1 infected cells inhibits cell carcinogenicity. These results indicate that the dysregulation of the Wnt signaling pathway via HTLV1 Tax and HBZ may be related to adult T-cell leukemia. Furthermore, it is observed that Tax through activation of NF-ĸB signaling pathway induced miR-146a expression in T-cell lines increases the growth of HTLV-1-infected cells (41). Although HIV doesn’t trigger tumorigenicity directly, it creates the conditions for many tumors to be activated through a weakened immune system and immune deficiency (91-95). Moreover, studies have shown that HIV proteins, such as Tat and nef, can interact with many pathways and cell regulatory networks (96-99). In spite of the fact that interactions (Figure 1) between the Wnt / β-catenin signaling pathways and HIV do not cause tumorigenicity, they have intense effects on HIV-caused neuropathogenesis (100). Weiser *et al. *(2013) showed that interactions between the nef virus and β-catenin proteins resulted in T-cell transmigration (45). Results of an *in vitro* study on the BV2 cells (a murine-derived microglial cell line) suggested that HIV infection and HIV-1 gp120 cause accumulation of Wnt3a and β-catenin and s activation of the Wnt/β-catenin signaling pathway (46). In another study, it was observed that the Tat protein interacts with TCF4 and inhibits Wnt signaling (43). Furthermore, Weiser reports a confirmed interaction between HIV nef and β-catenin (44). 

*Human papillomaviruses (HPVs)*

HPV is classified in *Papillomaviridae* (wart viruses) family. These viruses are equipped with several oncoproteins and are associated with some malignancies in humans and other species (101, 102). Many studies have shown that two HPV oncoproteins E6 and E7 are associated with the pathogenesis of cellular tumorigenesis. These proteins interact with two tumor suppressors Rb and P53, respectively (103, 104). Alteration of the wnt signaling by HPV plays an important role in cervical cancer (Figure 1). Various reports have revealed that oncoproteins E6 and E7 can lead to dysregulation of the wnt signaling pathways. One of the mechanisms by which HPV E6/E7 oncoproteins can modulate the Wnt pathway is regulation of SIAH-1-dependent ubiquitin/proteasome pathway for β-catenin degradation (47, 49, 105). During an *in vivo* study, it was observed that HPV16 E6 via interaction with Dvl2 can lead to activation of the canonical Wnt/β-catenin pathway in skin epidermis (48). Evidence suggests that E6 together with E6AP or ubiquitin-protein ligase E3A (an enzyme that is involved in targeting proteins for proteasomal degradation within cells) prevent β-catenin proteasomal degradation (51). Suppressing the expression of two viral oncogenes E6 and E7 reduces the β-catenin level and the re-expression of these two important viral oncogenes increases the β-catenin-TCF/ LEF-mediated transcription (49). In a mouse model, it was demonstrated that HPV16 E6 can stimulate beta-catenin expression and the two cellular proto-oncogenes MYC and CCND1 in skin cells (50). The profiling of genes induced by HPV18, such as the MYC, FZD, DKK, and WNT16 genes, has determined that HPV E6 can lead to the dysregulation of the Wnt pathway (52).

*Other viruses*

The cytomegalovirus (CMV) is a member of the *Herpesviridae* DNA virus family and the beta virus genus. CMV causes latent viral infections in host lymphoid tissues that can retain lifelong latent infections. CMV is a very common virus such that 40 to 80% of the world’s population are infected with CMV, which is usually asymptomatic. CMV, especially in immunocompromised individuals, can cause serious clinical complications such as retinitis, hepatitis, pneumonia, colitis, and encephalitis, which may lead to high mortality. Infants with infected mothers can develop congenital infections (106, 107).

Although a direct association between CMV and carcinogenicity has not been found, the evidence suggests that the virus can interfere with cellular pathways such as the Wnt signaling pathway (54, 108). The overexpression of the CMV-encoded chemokine receptor US28 in the transgenic mouse model has been shown to promote intestinal adenomatosis and accumulation of CCND1 and nuclear β-catenin. Further analyses have shown that US28 may increase β-catenin via the Rho-ROCK pathway (55). Another mechanism for the modulation of the Wnt pathway by CMV is the targeting of Wnt proteins via encoded viral miRNAs that target β-catenin (58). 

In addition, dysregulated Wnt signaling results in impurities in the fetus and its growth (108). Studies have also shown CMV US28 can upregulate Wnt2 and WISP2, and can downregulate Wnt5a/β, LRP6, CCND1, MYC, and DKK (56, 57).

Adenoviruses (members of the *Adenoviridae* family), containing a double strand DNA genome, have different viral groups. Adenoviruses infect both humans and animals (109). Various studies in animal models, especially hamsters and primates have shown that some viral groups in this large family can be carcinogenic and some of the proteins in the family have an oncoprotein role and can cause the alteration of the Wnt pathway (59-61). It has been observed that type-71 enteroviruses can cause the dysregulation of the Wnt pathway through the regulated expression of miRNAs (62). Additionally, it was shown that coxsackievirus miR-126 may target LRP6 (a key frizzled co-receptor component that is involved in the canonical Wnt pathway) and WRCH1 (a small signaling G protein that is involved in the non-canonical Wnt pathway) and promote β-catenin degradation (63).

## Conclusion

In this review, the effects of well-known human oncogenic viruses, as well as viruses that may indirectly contribute to carcinogenesis in the Wnt signaling pathway were evaluated and discussed. The results obtained from extended studies about the oncoproteins of these viruses and their interactions with the key Wnt signaling pathway indicate that most of these viruses have at least 1-2 oncoproteins and have clarified their mechanisms of action on the Wnt signaling pathway. Commonly, many of these interactions act through mechanisms such as modulation in the cytoplasmic and nuclear accumulation of β-catenin through direct and indirect targeting of the Wnt pathway proteins, inducing or coding the effective cellular and viral miRNAs on the Wnt pathway, epigenetic changes in the genes encoding the Wnt pathway including CTNNB1 gene and coding Wnt genes, silencing of SFRP proteins (antagonists of the Wnt signaling), prevention of β-catenin proteasomal degradation via interaction with the destruction complex proteins such as GSK3β and APC.

Taken together, according to the mentioned mechanisms and analysis of the results it seems that oncogenic viruses have common evolutionary mechanisms for modulation and interaction with the Wnt signaling pathway especially canonical pathway, which results in carcinogenesis of the tissue involved. Therefore, it is seen that interactions between viral oncoproteins and the Wnt signaling pathway potentially have an intense role in carcinogenesis, which can provide new insights into the pathogenesis of viral cancers and possible therapeutic strategies. However, more precise molecular mechanisms of these interactions and their role in viral carcinogenesis need to be further studied.
